# Long-Term Stabilization after Thoracic Endovascular Aortic Repair for a Behçet-Related Thoracic Aortic Aneurysm: A Case Report

**DOI:** 10.70352/scrj.cr.26-0577

**Published:** 2026-10-24

**Authors:** Kensuke Oue, Moe Kinoshita, Shintaro Okuda, Nobuo Kondo

**Affiliations:** Department of Cardiovascular Surgery, Kochi Health Sciences Center, Kochi, Kochi, Japan

**Keywords:** Behçet disease, thoracic aortic aneurysm, thoracic endovascular aortic repair, endoleak, immunosuppressive therapy, long-term follow-up

## Abstract

**INTRODUCTION:**

Behçet disease is a relapsing systemic vasculitis that can rarely cause aortic aneurysms or pseudoaneurysms. Endovascular repair is increasingly used for Behçet-related aortic lesions, but the long-term interaction between aneurysm remodeling, endoleak, and systemic inflammatory activity remains clinically important.

**CASE PRESENTATION:**

A 34-year-old man presented with persistent back pain due to an impending rupture of a newly developed saccular descending thoracic aortic aneurysm. Emergency thoracic endovascular aortic repair was performed before Behçet disease was definitively diagnosed. Postoperatively, persistent fever, elevated inflammatory markers, recurrent oral aphthous ulcers, and intestinal ulcerations led to the diagnosis of Behçet disease. The aneurysm initially regressed after corticosteroid therapy but rapidly re-expanded during steroid tapering, in parallel with fever, back pain, and increased C-reactive protein and erythrocyte sedimentation rate, despite no demonstrable type I or III endoleak. Additional endovascular reinforcement and intensified immunosuppressive therapy were performed. A type II endoleak from the left first lumbar artery was subsequently detected, but the aneurysm continued to regress under inflammatory control. More than 13 years after the initial repair, the treated aneurysm remains completely regressed.

**CONCLUSIONS:**

This case suggests that sac enlargement after endovascular repair for a Behçet-related aortic aneurysm should be evaluated as a potentially multifactorial event, including occult endoleak, persistent sac pressurization, stent-graft-related mechanical factors, and systemic inflammatory activity. In this case, durable stabilization was observed following combined endovascular treatment and sustained immunosuppressive therapy.

## Abbreviations

CRP
: C-reactive protein
ESR
: erythrocyte sedimentation rate
EULAR
: European League Against Rheumatism
MPR
: multiplanar reconstruction
PSL
: prednisolone
TEVAR
: thoracic endovascular aortic repair

## INTRODUCTION

Behçet disease is a chronic, relapsing, systemic inflammatory disorder characterized by recurrent oral aphthous ulcers, genital ulcers, skin lesions, and ocular involvement.^1)^ Vascular involvement may affect both venous and arterial systems, although venous thrombosis is more common.^1,2)^ Arterial lesions, particularly aortic aneurysms and pseudoaneurysms, are uncommon but potentially life-threatening because of rapid progression and rupture.^2,3)^

Open surgical repair has traditionally been used for Behçet-related aortic aneurysms; however, postoperative anastomotic pseudoaneurysm formation and recurrent aneurysmal disease remain major concerns.^4–6)^ Endovascular repair avoids direct suturing of inflamed arterial tissue and has increasingly been used as a less invasive alternative, particularly in combination with immunosuppressive therapy.^5–9)^ We report a patient with a Behçet-related descending thoracic aortic aneurysm in whom aneurysm behavior closely paralleled systemic inflammatory activity, followed by complete regression over more than 13 years after TEVAR and sustained immunosuppressive therapy.

## CASE PRESENTATION

A 34-year-old man presented with persistent back pain. His medical history was significant for chronic pancreatitis. A contrast-enhanced CT scan performed 1 year earlier during treatment for pancreatitis showed no evidence of aortic aneurysm. In March 2013, he visited a local clinic because of back pain and was initially treated conservatively. However, his symptoms persisted, and repeat CT imaging in April revealed a newly developed saccular aneurysm of the descending thoracic aorta near the thoracoabdominal junction (**Fig. 1A**–**1C**). He was referred to our institution with suspected impending rupture.

**Fig. 1:**
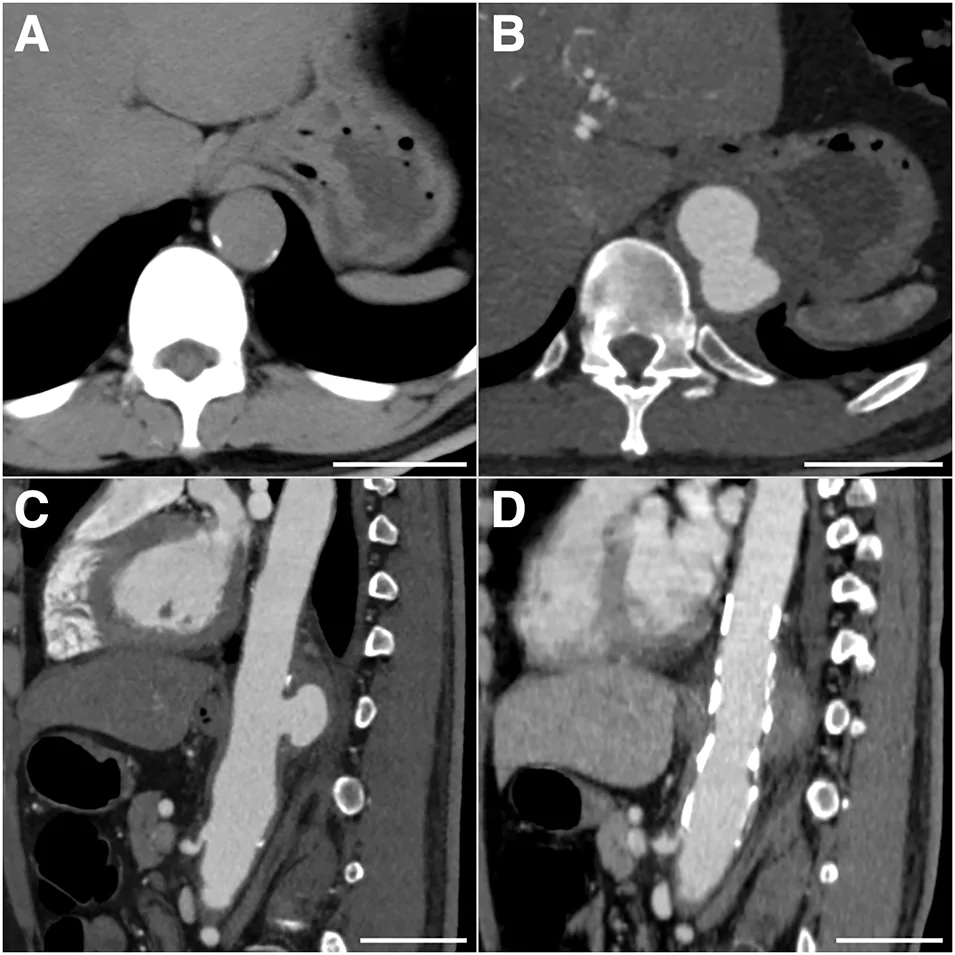
CT findings before and after the initial TEVAR. (**A**) CT obtained 1 year before admission showing no aortic aneurysm. (**B**) Axial contrast-enhanced CT at presentation showing a newly developed saccular aneurysm of the descending thoracic aorta. (**C**) Sagittal MPR image showing the aneurysm near the thoracoabdominal junction. (**D**) Postoperative sagittal MPR image after the initial TEVAR showing successful exclusion of the aneurysm. MPR, multiplanar reconstruction; TEVAR, thoracic endovascular aortic repair

On admission, his height was 182 cm, body weight was 79.7 kg, blood pressure was 127/66 mmHg, heart rate was 93 beats/min, and body temperature was 37.2°C. Laboratory tests showed leukocytosis (13650/μL) and an elevated CRP level (3.89 mg/dL). Renal and hepatic functions were within normal limits.

Because the aneurysm was symptomatic and had developed rapidly, emergency TEVAR was performed before an infectious aneurysm could be definitively excluded. Distal extension stent grafts (Zenith TX2; Cook Medical, Bloomington, IN, USA; 32 × 80 mm and 34 × 77 mm) were selected to cover the localized saccular lesion while limiting unnecessary aortic coverage. The proximal landing zone was in the descending thoracic aorta (diameter, 28 mm), and the distal landing zone was just above the celiac artery (diameter, 30 mm). Total longitudinal coverage was approximately 120 mm. Postoperative CT showed successful aneurysm exclusion without endoleak (**Fig. 1D**). Access was obtained by right common femoral artery cut-down, and no access-site pseudoaneurysm or infection occurred.

From POD 2, the patient developed persistent fever exceeding 39°C and marked elevation of inflammatory markers. Because infectious aortitis remained in the differential diagnosis, empiric broad-spectrum antimicrobial therapy was administered, including cefazolin, meropenem, ciprofloxacin, piperacillin/tazobactam, and vancomycin. However, repeated blood cultures remained negative, and no causative organism was identified. Follow-up CT showed no findings suggestive of infectious aortitis or graft infection, such as perigraft abscess, gas formation, or progressive periaortic soft-tissue changes. On POD 19, recurrent oral aphthous ulcers appeared, and antimicrobial therapy was discontinued.

Abdominal pain subsequently developed. CT demonstrated inflammatory changes in the colon, and colonoscopy revealed multiple ulcerative lesions in the ileum. After exclusion of infectious etiologies, including cytomegalovirus infection and tuberculosis, Behçet disease was diagnosed on POD 38. PSL at 50 mg/day was started, resulting in rapid resolution of fever and improvement in inflammatory markers.

After steroid initiation, follow-up CT showed marked aneurysm regression. By POD 83, the aneurysm sac diameter had decreased from 47 to 40 mm (**Fig. 2A**). On POD 76, the CRP level was 0.80 mg/dL and the ESR was 12 mm/h. PSL was started at 50 mg/day on POD 38. After fever, back pain, CRP, and ESR improved, the dose was reduced to 25 mg/day on POD 75. During this reduced-dose period, high-grade fever and recurrent back pain developed, accompanied by re-elevation of inflammatory markers. On POD 108, CT demonstrated rapid aneurysm re-expansion from 40 to 73 mm (**Fig. 2B**). This enlargement was accompanied by recurrent back pain, fever, and marked elevation of inflammatory markers, with a CRP level of 9.10 mg/dL and an ESR of 74 mm/h. No definite type I or type III endoleak was detected on CT or angiography. However, because the aneurysm had rapidly enlarged and had become symptomatic again, we considered the lesion to be at high risk of imminent rupture. Sagittal MPR showed localized enlargement around the previously treated aneurysmal segment (**Fig. 2C**).

**Fig. 2:**
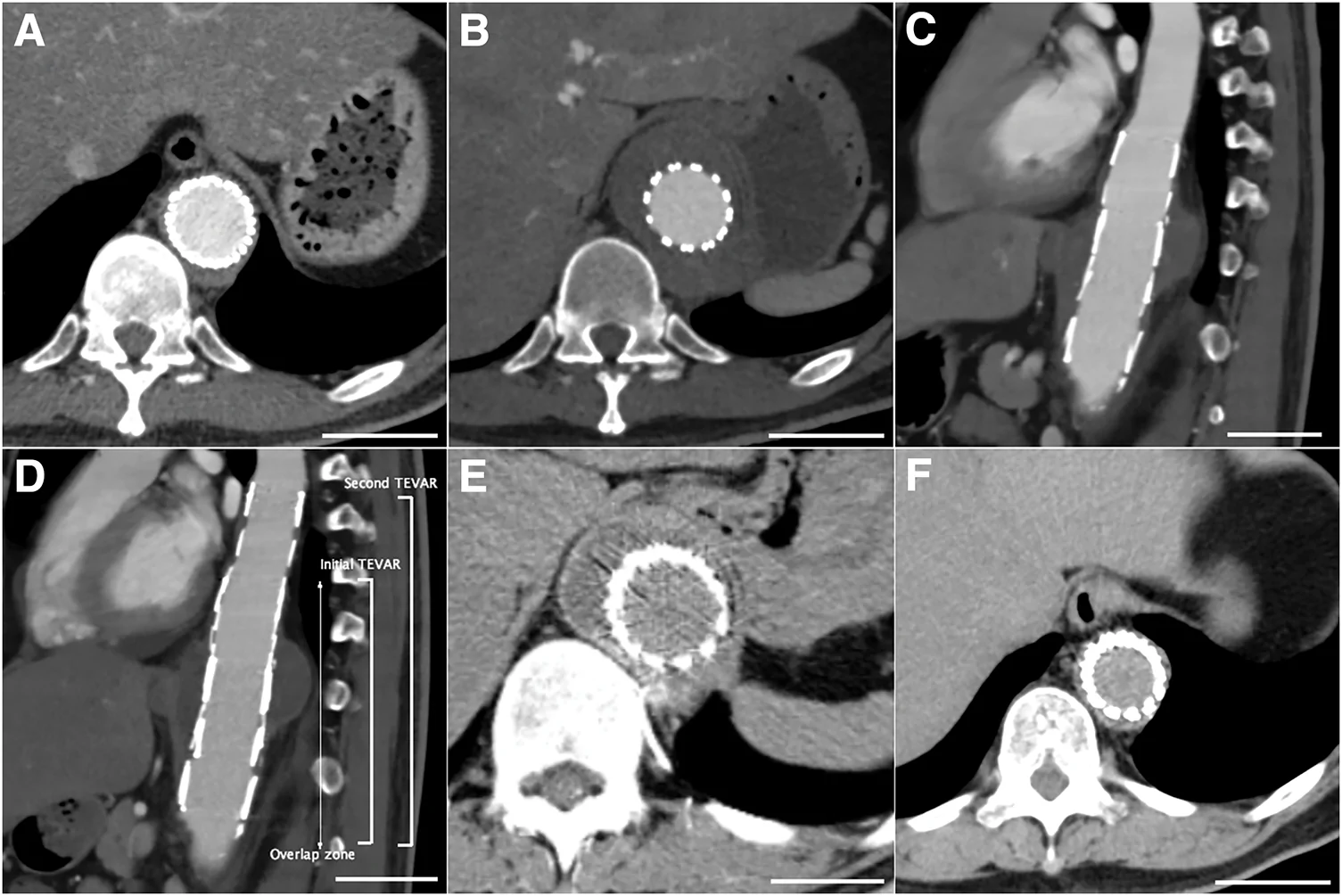
CT findings during the clinical course after TEVAR. (**A**) CT on POD 83 showing regression of the aneurysm sac from 47 mm to 40 mm after initiation of corticosteroid therapy. (**B**) Rapid aneurysmal re-expansion to 73 mm on POD 108 during steroid tapering. (**C**) Sagittal MPR showing localized enlargement of the treated aneurysmal segment. (**D**) CT after the second TEVAR. Brackets indicate the initial and second TEVAR constructs, and the double-headed arrow indicates the overlap zone. (**E**) Contrast-enhanced CT obtained during selective angiography of the left first lumbar artery showing a type II endoleak. (**F**) Long-term follow-up CT showing complete regression of the treated aneurysm. MPR, multiplanar reconstruction; TEVAR, thoracic endovascular aortic repair

On POD 108, PSL was increased to 50 mg/day, azathioprine at 50 mg/day was initiated, and trimethoprim-sulfamethoxazole was started to prevent opportunistic infection. Azathioprine was selected as a steroid-sparing immunosuppressive agent after multidisciplinary discussion. This choice was made because the acute aneurysmal lesion had already been mechanically excluded by emergency TEVAR, intestinal involvement was present, and long-term maintenance immunosuppressive therapy was considered necessary. Blood cultures obtained during this inflammatory flare remained negative. On POD 112, additional TEVAR was performed for mechanical stabilization of the rapidly enlarging aneurysmal segment. The procedure was not intended to treat a proven type I or type III endoleak, because neither CT nor angiography showed a definite sealing failure or component disruption. Definite stent-graft migration or obvious inadequate apposition was also not identified. Rather, because the initial repair had been performed using 2 relatively short distal extension components to limit aortic coverage, the second TEVAR was intended to reinforce the overlap between the initial stent-graft components and provide additional internal support to the inflamed aneurysmal aortic segment. A Zenith TX2 TAA Endovascular Graft (ZTEG-2P-30-140-PF; Cook Medical) and a distal extension graft (ESBE-34-77-T-PF; Cook Medical) were used. The main body was deployed with its distal end 1 stent segment proximal to the distal end of the initial graft, and the extension graft was placed to align its lower edge with the distal end of the initial construct. This configuration was selected to increase component overlap and provide additional mechanical support to the previously treated aneurysmal segment (**Fig. 2D**).

The clinical course, inflammatory markers, immunosuppressive therapy, and aneurysm size are summarized in **Fig. 3**. On POD 120, selective angiography of the left first lumbar artery and CT obtained during this procedure demonstrated a type II endoleak arising from the left first lumbar artery (**Fig. 2E**). No type I or type III endoleak had been identified at the time of rapid aneurysmal re-expansion. Because the aneurysm sac gradually decreased in size despite the type II endoleak, conservative management was selected. At discharge on POD 172, the sac diameter had decreased to 35 mm, with normalization of inflammatory markers (ESR 5 mm/h, CRP 0.09 mg/dL). PSL had been reduced to 25 mg/day by discharge.

**Fig. 3:**
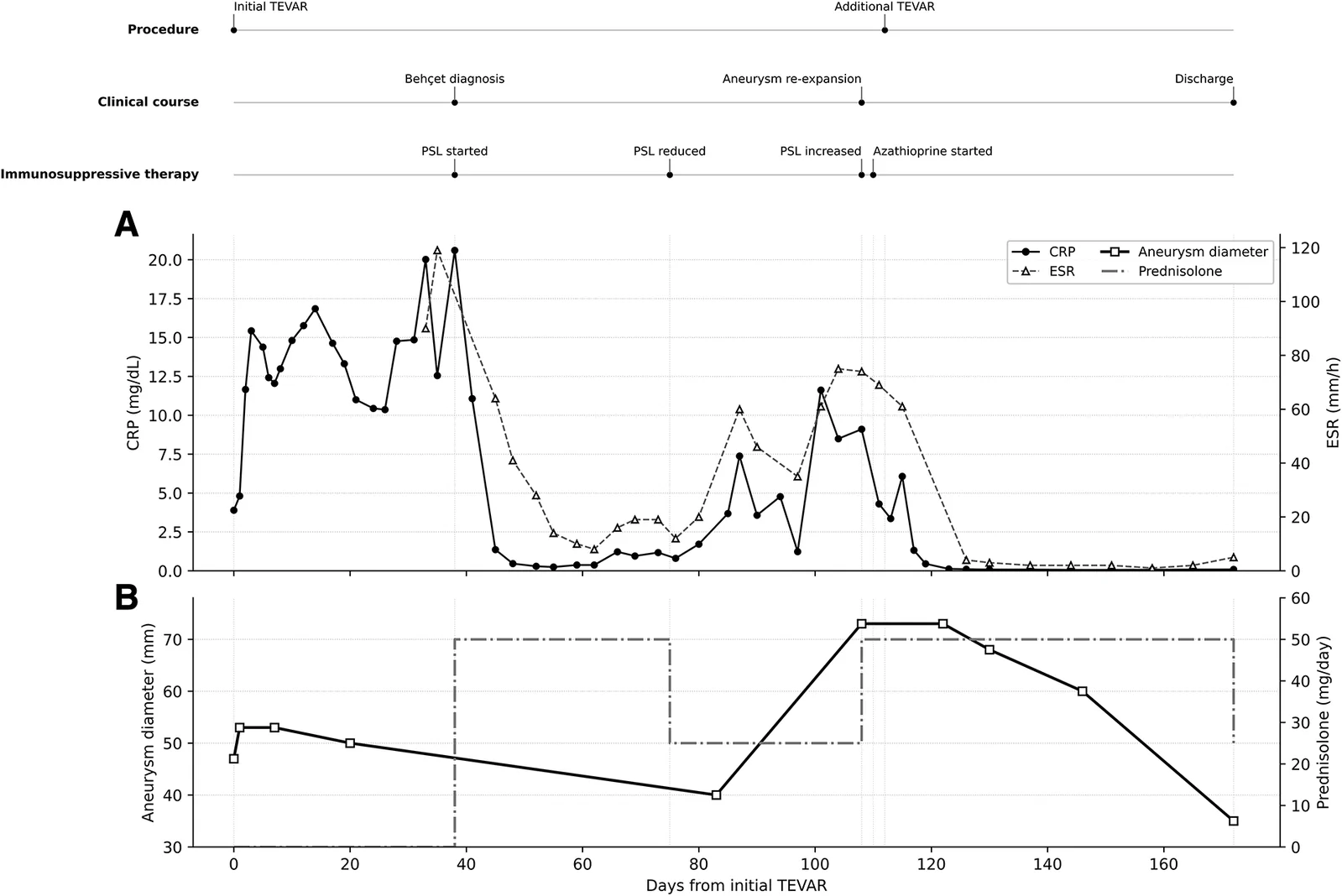
Clinical course during the initial hospitalization. (**A**) Aneurysm sac diameter, CRP, and ESR are shown with key clinical events. (**B**) PSL dose changes during hospitalization. CRP, C-reactive protein; ESR, erythrocyte sedimentation rate; PSL, prednisolone; TEVAR, thoracic endovascular aortic repair

After discharge, PSL was tapered stepwise while monitoring symptoms, CRP, ESR, and CT findings. The dose was reduced to 20 mg/day on POD 208, 17.5 mg/day on POD 236, 15 mg/day on POD 320, 12.5 mg/day on POD 355, and 10 mg/day by approximately 1 year. It was subsequently maintained at 7.5 mg/day from POD 873 onward, together with azathioprine at 50 mg/day. During long-term follow-up, systemic inflammatory activity remained generally controlled, with CRP levels ranging from approximately 0.09 to 2.15 mg/dL and ESR ranging from approximately 2 to 32 mm/h after discharge. No clinical relapse of Behçet disease requiring escalation of immunosuppressive therapy occurred.

After discharge, CT surveillance was performed every 6 months during the first year and annually thereafter to evaluate the treated aneurysmal segment, endoleak status, and untreated aortic segments. Serial CT surveillance showed no recurrent enlargement of the treated aneurysmal segment, and complete regression was maintained during follow-up exceeding 13 years (**Fig. 2F**). The maximum short-axis diameter of the non-stented descending aorta between the distal end of the stent graft and the celiac artery origin was measured on axial or MPR CT images. This diameter increased gradually from 33.9 mm at presentation to 38.6 mm at 1 year and 44.8 mm over 13 years, without rapid expansion or periaortic inflammatory change. PSL was not increased solely for the gradual enlargement of this segment, and lifelong imaging surveillance was continued.

## DISCUSSION

This case illustrates the complex relationship between mechanical factors and systemic inflammatory activity during the long-term course of a Behçet-related descending thoracic aortic aneurysm after TEVAR. The principal educational message is that rapid sac re-expansion should be interpreted as a potentially multifactorial event, while long-term stabilization in this patient was observed following combined endovascular treatment, sustained immunosuppressive therapy, and continued imaging surveillance.

Arterial involvement is one of the most serious manifestations of Behçet disease.^1,2)^ Aortic aneurysms and pseudoaneurysms may progress rapidly and rupture.^2,3)^ In the present case, a saccular descending thoracic aortic aneurysm developed within 1 year and was associated with persistent back pain, suggesting impending rupture. Infectious aortitis was ultimately considered unlikely because repeated blood cultures were negative, no causative organism was identified, serial CT showed no perigraft abscess, gas formation, or progressive periaortic inflammatory changes, and the systemic manifestations were consistent with Behçet disease. The rapid clinical response to corticosteroid therapy and the absence of graft infection during more than 13 years of follow-up further supported a noninfectious inflammatory etiology.

The decision to perform TEVAR was also supported by the known limitations of open repair in Behçet-related arterial disease. Kwon et al. reported recurrent aneurysms in 6 of 12 patients treated for abdominal aortic aneurysms associated with Behçet disease, with an overall recurrence rate of 38.1% per procedure.^4)^ Yin et al. summarized previous reports of postoperative anastomotic pseudoaneurysm formation after open vascular reconstruction, with rates ranging from 12.9% to 50%, and observed no anastomotic pseudoaneurysm after stent-graft implantation in their own cohort.^6)^ These findings support the value of avoiding direct anastomosis to inflamed arterial tissue when endovascular repair is anatomically feasible.

The indication for the second TEVAR requires careful interpretation. At the time of re-expansion, neither CT nor angiography demonstrated a type I or III endoleak. Therefore, the second TEVAR was not performed as treatment for a proven sealing failure or component disruption. Definite stent-graft migration or obvious inadequate apposition was also not identified. Instead, the indication was based on the rapid increase in sac diameter from 40 to 73 mm, recurrent back pain, and active systemic inflammation, which together suggested a high risk of rupture. The portion considered vulnerable was the previously treated aneurysmal segment, including the overlap zone between the 2 short distal extension components used during the initial TEVAR. Because the initial repair consisted of 2 short distal extension components, we considered that additional stent-graft reinforcement could increase component overlap and provide internal support to the inflamed aneurysmal aortic segment. However, this approach should be interpreted as an individualized and empirical treatment strategy under an urgent clinical situation, rather than as an established mechanical stabilization strategy for Behçet-related aneurysmal re-expansion. The mechanical benefit of additional internal support cannot be established from this single case, and the effects of additional radial force on an inflamed and fragile aortic wall are difficult to predict.

Liu et al. reported recurrent pseudoaneurysms in 2 patients who did not adhere to immunosuppressive therapy after endovascular treatment, including 1 death due to rupture.^7)^ Balcioglu et al. reported no aneurysm recurrence during a mean follow-up of 40 months after endovascular repair combined with adjunctive immunosuppressive therapy.^5)^ More recent studies have also supported the feasibility of endovascular repair while emphasizing that recurrent pseudoaneurysm, endoleak, and reintervention remain important late events.^8,9)^

The most distinctive aspect of the present case was rapid sac re-expansion during steroid tapering without a demonstrable type I or III endoleak. This finding should be interpreted cautiously. On POD 120, a type II endoleak arising from the left first lumbar artery was identified by selective angiography and CT obtained during this procedure. Therefore, occult or intermittent low-flow type II endoleak and persistent sac pressurization may have contributed to aneurysmal enlargement. However, the later detection of a type II endoleak does not prove that the same endoleak was already present at the time of the initial rapid re-expansion on POD 108. At the same time, sac re-expansion was temporally associated with fever, recurrent back pain, and marked increases in CRP and ESR. Systemic inflammatory activity may therefore have contributed to aneurysmal instability, but causality cannot be established, and its relative contribution cannot be determined from this single case. Because intensified immunosuppressive therapy and the second TEVAR were performed almost simultaneously, the individual contribution of each intervention to subsequent aneurysm regression cannot be distinguished. Long-term stabilization was observed following combined endovascular treatment and sustained immunosuppressive therapy.

The 2018 EULAR recommendations state that medical treatment with corticosteroids and cyclophosphamide is necessary before repair of aortic and peripheral arterial aneurysms when circumstances permit, but surgery or stenting should not be delayed if the patient is symptomatic.^10)^ In the present case, emergency TEVAR was required before Behçet disease was diagnosed because of persistent back pain and rapid aneurysm development suggesting impending rupture. After the diagnosis was established, azathioprine was selected as a steroid-sparing maintenance agent after multidisciplinary discussion. This decision was not based on a fixed institutional protocol, but on the clinical context: emergency TEVAR had already been performed, intestinal involvement was present, and long-term immunosuppressive maintenance therapy was required. Therefore, azathioprine in this case should be interpreted as individualized steroid-sparing maintenance therapy rather than standard induction therapy for Behçet-related aortic aneurysm.

The subsequent inflammatory flare during PSL dose reduction indicated that remission had not yet been stabilized. Therefore, tapering of immunosuppressive therapy in vascular Behçet disease should be individualized and accompanied by close clinical, laboratory, and imaging surveillance.

The more than 13-year follow-up is an important feature of this report. After discharge, long-term management consisted of maintenance azathioprine therapy, low-dose PSL, and scheduled CT surveillance. The treated aneurysm has remained completely regressed, suggesting that durable remodeling is possible after successful aneurysm exclusion and sustained inflammatory control. Conversely, the non-stented distal descending aortic segment enlarged gradually from 33.9 to 44.8 mm over 13 years. This enlargement occurred without rapid expansion, symptoms, marked inflammatory relapse, or periaortic inflammatory changes, and PSL was not increased solely for this lesion. We therefore considered continued surveillance to be appropriate. However, this segment cannot be regarded as completely stable. Lifelong imaging follow-up is required, and additional treatment would be considered if rapid enlargement, symptoms, new periaortic inflammatory changes, or further size progression suggesting increased rupture risk develops.

This report has limitations. It is a single case, and the mechanism of sac re-expansion cannot be definitively determined. The relative contributions of occult type II endoleak, sac pressurization, stent-graft configuration, and inflammatory wall vulnerability remain uncertain. Despite these limitations, the long-term course provides a practical lesson: in Behçet-related aortic aneurysm, successful endovascular exclusion should be integrated with sustained control of systemic vasculitis and long-term surveillance of both treated and untreated aortic segments.

## CONCLUSIONS

TEVAR provided effective emergency exclusion of a symptomatic Behçet-related thoracic aortic aneurysm. Subsequent aneurysmal re-expansion occurred in association with systemic inflammatory recurrence, but occult type II endoleak and persistent sac pressurization may also have contributed. Durable regression was observed following additional endovascular reinforcement and intensified immunosuppressive therapy. This case highlights the importance of considering both mechanical and inflammatory factors in the management of Behçet-related aortic aneurysms and of maintaining lifelong imaging surveillance.

## DECLARATIONS

### Use of artificial intelligence–assisted tools

Artificial intelligence–assisted tools (ChatGPT, OpenAI) were used for language editing and improving the clarity of the manuscript. The authors critically reviewed and revised the content and took full responsibility for the final version of the manuscript.

### Availability of data and materials

All data supporting the conclusions of this article are included within the article.
